# GPCS Stratification of Exercise-Induced Gut Microbiota and Metabolome Remodeling in IBS: An Exploratory Multi-Omics Study

**DOI:** 10.3390/nu18121972

**Published:** 2026-06-18

**Authors:** Francesco Maria Calabrese, Antonella Bianco, Margherita Chiarini, Laura Prospero, Isabella Franco, Matteo Bernardi, Giuseppe Celano, Maria Calasso, Giuseppe Riezzo, Nicola Verrelli, Benedetta D’Attoma, Antonia Ignazzi, Carmen Aurora Apa, Gianluigi Giannelli, Maria De Angelis, Francesco Russo

**Affiliations:** 1 Department of Soil, Plant and Food Science, University of Bari Aldo Moro, 70126 Bari, Italy; margherita.chiarini@uniba.it (M.C.); matteo.bernardi@uniba.it (M.B.); giuseppe.celano@uniba.it (G.C.); maria.calasso@uniba.it (M.C.); carmen.apa@uniba.it (C.A.A.); maria.deangelis@uniba.it (M.D.A.); 2 Laboratory of Movement and Wellness, National Institute of Gastroenterology IRCCS “Saverio de Bellis”, Castellana Grotte, 70013 Bari, Italy; antonella.bianco@irccsdebellis.it (A.B.); laura.prospero@irccsdebellis.it (L.P.); nicola.verrelli@irccsdebellis.it (N.V.); 3 Functional Gastrointestinal Disorders Research Group, National Institute of Gastroenterology IRCCS “Saverio de Bellis”, Castellana Grotte, 70013 Bari, Italy; isabella.franco@irccsdebellis.it (I.F.); giuseppe.riezzo@irccsdebellis.it (G.R.); benedetta.dattoma@irccsdebellis.it (B.D.); antonia.ignazzi@irccsdebellis.it (A.I.); 4 Scientific Direction, National Institute of Gastroenterology IRCCS “Saverio de Bellis”, Castellana Grotte, 70013 Bari, Italy; direzionescientifica@irccsdebellis.it

**Keywords:** low-mild IBS, GPCS, 16S rRNA gene sequencing, untargeted metabolomics, targeted metabolomics

## Abstract

Background/Objectives: Exercise is increasingly recognized as a modulator of host–microbiome interactions, yet its role in irritable bowel syndrome (IBS) remains poorly characterized. Methods: In this prospective, single-arm, before-and-after interventional study, we used an integrated multi-omics approach based on metataxonomics and metabolomics to assess the effects of a structured 12-week moderate aerobic exercise program in 80 patients with mild-to-moderate IBS, stratified by Global Physical Capacity Score (GPCS). Biochemical and inflammatory markers have been gathered. Results: Exercise did not alter overall microbial diversity but selectively enriched short-chain fatty acid (SCFA)-producing taxa and remodeled the volatile organic compound (VOC) profile toward a more efficient metabolic state. Notably, conventional biochemical and inflammatory markers failed to distinguish response subgroups, whereas GPCS stratification revealed distinct microbial and metabolomic trajectories. Individuals with higher baseline physical capacity had higher acetate levels and lower levels of VOCs associated with dysbiosis and oxidative stress. Conclusions: Our results suggest that baseline physical capacity is a primary determinant of the microbiome’s responsiveness to exercise, challenging the reliance on static biochemical profiling. Despite the lack of a control group and the exploratory nature of some metabolomic signals, this study provides a framework for precision exercise interventions in IBS. Our work identifies GPCS as a clinically relevant stratification tool. The full trial protocol is registered on ClinicalTrials.gov under the identifier NCT05453084.

## 1. Introduction

Irritable bowel syndrome (IBS) is among the most prevalent functional gastrointestinal disorders worldwide, with an estimated prevalence ranging from 3% to 9% in the adult population [1,2]. Its pathophysiology is multifactorial, including key determinants such as altered intestinal motility, visceral hypersensitivity, dysregulation of the gut–brain axis, low-grade mucosal inflammation, and, increasingly, perturbations of the gut microbiota [3,4]. The latter is frequently associated with altered metabolic profiles, characterized by reduced production of short-chain fatty acids (SCFAs), notably butyrate, propionate and acetate, and by the accumulation of pro-inflammatory metabolites and volatile organic compounds (VOCs) indicative of a dysbiotic fermentation [5,6]. Collectively, these alterations contribute to the persistence of chronic symptoms and a marked impairment in the quality of life [7].

Current therapeutic strategies for IBS often provide only partial symptomatic relief and do not substantially modify the natural history of this disease. Pharmacological interventions, including antispasmodics, laxatives and antidiarrheals, as well as probiotics and exclusion diets, such as the low-FODMAP regimen, display a non-homogeneous efficacy, limited long-term adherence and non-negligible economic and social costs [8]. Within this context, regular physical activity has emerged as a safe, sustainable, and potentially pleiotropic non-pharmacological intervention capable of simultaneously targeting multiple pathophysiological pathways [9,10].

Growing evidence indicates how moderate aerobic exercise may ameliorate IBS symptomatology through several mechanisms, including improved intestinal motility, reduced visceral sensitivity, neuroendocrine modulation of the gut–brain axis, and, notably, regulation of gut microbiota commensal composition and related functions [11,12]. Recent studies have shown that regular physical activity promotes the enrichment of SCFA-producing bacterial taxa, particularly within the Firmicutes and Bacteroidetes phyla, and shifts the fecal VOC profile toward more efficient microbial metabolism [13,14]. However, inter-individual responses to exercise remain heterogeneous, suggesting the existence of biologically distinct subgroups with varying degrees of physiological plasticity [9].

This heterogeneity poses a clinically relevant challenge: how can patients with IBS who are most likely to benefit from exercise as a stand-alone intervention be identified before prescription, without the need for adjunctive pharmacological or dietary strategies? The development of a simple, reliable functional stratification tool would represent a critical step toward personalized exercise-based interventions for IBS management.

In this context, our group has developed the Global Physical Capacity Score (GPCS), a composite index integrating cardiorespiratory fitness, muscular strength, and flexibility, expressed on a continuous scale and categorized into three functional groups, i.e., below-average, on-average, and above-average [15,16]. The GPCS has demonstrated the ability to stratify populations with functional disorders, including IBS and fibromyalgia, into subgroups characterized by distinct patterns of intestinal barrier dysfunction and differential physiological responses to exercise [17,18]. Unlike conventional biochemical markers, the GPCS captures a functional dimension of health baseline physical capacity that is not reflected in standard blood tests and may represent a key determinant of adaptive trajectories to exercise.

The present study aims to evaluate the effects of a moderate aerobic exercise program in a cohort of patients with mild-to-moderate IBS by examining fecal microbiota composition (16S rRNA metataxonomics), metabolomic profiles (targeted SCFAs and untargeted VOCs), inflammation markers, and intestinal permeability. Specifically, we assess how GPCS stratification better stratified samples than the physical activity treatment (pre- and post-groups) based on differential microbial and metabolomic responses to the intervention, with direct implications for the clinical exercise prescription as a stand-alone therapy in IBS.

We hypothesize that moderate aerobic exercise induces selective modulation rather than global restructuring of the gut microbiota, marked by preferential enrichment of SCFA-producing taxa. Furthermore, we propose that this modulation is more pronounced and biologically coherent in individuals with higher baseline GPCS. If confirmed, these findings would support using the GPCS as a simple, direct clinical tool to guide exercise prescription in IBS, contributing to the development of a personalized, non-pharmacological therapeutic framework.

## 2. Materials and Methods

### 2.1. Study Design and Participants

This was a prospective, single-arm, before–after interventional study conducted at a single tertiary referral center. Patient specimen recruitment was conducted through a collaboration between the Research Group on Functional Gastrointestinal Disorders and the Laboratory of Epidemiology and Statistics at the IRCCS “Saverio de Bellis” National Institute of Gastroenterology in Castellana Grotte (Italy), as previously described [15]. The study took place from May 2022 to December 2023 and was registered on ClinicalTrials.gov under the accession number (ID: NCT05453084).

Adult participants were selected from the outpatient clinic for celiac disease and functional gastrointestinal disorders, diagnosed with irritable bowel syndrome (IBS) according to the Rome III or IV criteria, or referred by general practitioners. To be included, participants had to be aged 18–65, willing to participate in the structured walking group, and hold a medical certificate for non-competitive sports.

Participants were excluded if they had: significant cardiac, hepatic, neurological, or psychiatric conditions; gastrointestinal disorders other than IBS; a history of following low-FODMAP, vegan, or gluten-free diets; ongoing antidepressant treatment; significant orthopedic or neuromuscular limitations; or absolute contraindications to physical exercise. Additionally, participants who missed more than 20% of scheduled sessions were excluded from the analysis.

The study was conducted in accordance with the principles of the Declaration of Helsinki. The protocol was approved by the Comitato Etico IRCCS Istituto Oncologico “Gabriella Serio” Prot. 177/C.E. dated on the 13 May 2022.

The primary outcome of this study was the change in fecal microbiota composition, assessed by 16S rRNA metataxonomics (diversity matrices, and genus-level differential abundance), between baseline (T0, before the intervention) and post-intervention (T1, after 12 weeks). Secondary outcomes included: (i) changes in fecal short-chain fatty acid (SCFA) concentrations (acetic, butyric, propionic, isobutyric, and isovaleric acids) measured by targeted GC/MS; (ii) changes in the fecal volatile organic compound (VOC) profile assessed by untargeted GC/MS; (iii) changes in systemic inflammatory markers (cytokines, complete blood count); (iv) differential outcome patterns across GPCS stratification groups (below-average, on-average, and above-average). All primary and secondary outcomes were assessed at T0 and T1 using identical procedures and operators.

Sample size was estimated based on previously published data from our group [15,16], where a structured moderate aerobic exercise program produced a clinically meaningful improvement in IBS symptom scores (IBS-SSS) and physical performance parameters. Assuming a moderate effect size (Cohen’s *d* = [0.5]), a two-tailed alpha of 0.05, and a desired statistical power of 80%, a minimum of 68 participants was required. To account for an anticipated dropout rate of approximately 20% (defined as missing more than 20% of scheduled sessions), a target enrollment of 80 participants was established. Given the exploratory and multiomics nature of this study, no formal multiple-testing adjustment was applied to the sample size calculation.

Because of the nature of the supervised exercise intervention, blinding participants and exercise instructors was not feasible; the study was therefore conducted as an open-label trial. To minimize assessment bias, all laboratory analyses (including DNA extraction, 16S rRNA sequencing, GC/MS metabolomics, and biochemical assays) were performed by personnel blinded to participants’ GPCS group assignment and clinical features. Outcome assessors and bioinformatics analysts were also blinded to group allocation throughout the analytical pipeline.

### 2.2. Exercise Protocol: Assessment of Physical Fitness

Before the start of the program, each participant underwent three field functional tests to establish their baseline physical profile and calibrate the training intensity. Cardiorespiratory fitness was assessed using the 2 km walk test [19]; hand grip strength and spinal flexibility were measured using the Hand Grip test [20] and the Sit and Reach test [21], respectively. The same battery of tests was repeated at the end of the 12-week intervention to allow for a pre-post comparison.

To improve measurement reliability, three 60-min familiarization sessions were scheduled the week before the official assessment. On these occasions, the technical staff explained the correct procedures, corrected walking technique, recommended suitable footwear and checked that the heart rate monitors were working properly, whilst also addressing any queries from participants.

To minimize methodological variability, the conditions under which the tests were administered were kept constant across the two assessments: the same venue, the same operators, the same time and the same equipment.

### 2.3. Structure of the Physical Activity Program

The program was organized in the form of a ‘Walking Group’, with the following characteristics:

(i) Frequency. Outdoor sessions on an urban route, three times a week on non-consecutive days, for a total of 12 weeks.
(ii) Intensity. Moderate intensity, corresponding to 60–75% of individual maximum heart rate (calculated using the Tanaka formula), monitored in real time with a heart rate monitor. The Talk Test and the Borg Scale were used as additional indicators of respiratory effort and perceived fatigue.
(iii) Type. Walking at variable speeds, between 5 and 10 km/h.
(iv) Duration and structure of the session. Each session lasted 60 min (180 min/week). Supervision was provided by graduates in Science and Techniques of Preventive and Adapted Physical Activity; attendance was recorded at each session.

### 2.4. Global Physical Capacity Score

Overall physical capacity was assessed using a battery of motor tests validated in adults, designed to evaluate three main dimensions: cardiorespiratory endurance, strength and flexibility. The results of each test were converted into a score ranging from 1 to 5, based on pre-established reference tables differentiated by sex and age; the sum of the three scores generated a composite index with a theoretical range of 3–15 points.

The GPCS used in this study is based on the methodological model [22], which has already been used in several of our studies [15,18]. Compared to a single test, this composite index provides a more comprehensive assessment of physical performance, including components functionally relevant to activities of daily living.

### 2.5. DNA Extraction and Quantification of Integrity

Total DNA was extracted from each sample using the “FastDNA SPIN Kit for Soil” (MP Biomedicals, Inc, Santa Ana, CA, USA) and quantified by fluorimetry using the Qubit™ Flex Fluorometer (Invitrogen Co., Carlsbad, CA, USA).

After quantification, a series of qualitative and integrity tests were performed on the samples using the Agilent 4200 TapeStation System under the Genomic DNA ScreenTape kit (Agilent Technologies, Santa Clara, CA, USA).

### 2.6. 16S rRNA Gene Amplification and Library Preparation

Library preparation for 16S rRNA gene sequencing was performed according to the “16S Metagenomic Sequencing Library Preparation” protocol from Illumina. PCR reaction was prepared using 2.5 µL of microbial DNA (5 ng/µL), 5 µL of 1 µM forward adapter + primer (5′ TCGTCGGCAGCGTCAGATGTGTATAAGAGACA**GCCTACGGGNGGCWGCAG**, primer sequence in bold), 5 µL of 1 µM reverse adapter plus primer (5′ GTCTCGTGGGCTCGGAGATGTGTATAAGAGACAGG**ACTACHVGGGTATCTAATCC**, primer sequence in bold), and 12.5 µL of 2× KAPA HiFi HotStart ReadyMix (Hoffmann-La Roche AG, Basel, Switzerland), for a final volume of 25 µL per sample. The reaction was carried out using the following conditions: 95 °C hot start, denaturation for 3 min, followed by 25 cycles of 95 °C for 30 s, 55 °C for 30 s, and 72 °C for 30 s, followed by a final 72 °C extension for 5 min.

The index PCR step adds unique i5 and i7 indices, along with Illumina-compatible adapters, enabling the identification and demultiplexing of each sample after sequencing and the anchoring of fragments to the MiSeq flow cell during cluster generation. For each sample, the reaction was prepared by mixing 25 µL of 2× KAPA HiFi HotStart ReadyMix (Hoffmann-La Roche AG, Basel, Switzerland), with 5 µL of the Nextera XT Index 1 (N7XX) primer and 5 µL of the Nextera XT Index 2 (S5XX) primer from the Nextera XT Index Kit (FC-131-1001, Illumina, Inc., San Diego, CA, USA), and 10 µL of PCR-grade water. Lastly, 5 µL of each purified DNA library was added, bringing the total reaction volume to 50 µL. Amplification was performed in a thermal cycler, beginning with a denaturation step at 95 °C for 3 min, followed by 8 cycles of 95 °C for 30 s, 55 °C for 30 s, and 72 °C for 30 s. A 5-min extension at 72 °C completed the reaction, which is then stored at 4 °C until further analysis.

### 2.7. Library Validation, Quantification, Normalization, Pooling, Dilution and Denaturation

The quality and size of the obtained libraries were subsequently assessed using the D1000 assay on the Agilent 4200 TapeStation instrument (Agilent Technologies, Santa Clara, CA, USA).

The pooled library underwent denaturation and dilution. In parallel, a PhiX control library (v3) (Illumina, Inc., San Diego, CA, USA) was also prepared, denatured, and diluted to the same concentration. The NGS sequencing was carried out on a MiSeq2 sequencer with 2 × 250 bp paired-end runs (Illumina, Inc., San Diego, CA, USA).

### 2.8. Bioinformatics Analyses

FastQ files were pre-processed using the QIIME2 tool (v. 2025.10) within the Conda environment [23]. Specifically, demultiplexed paired-end reads were first imported, and Illumina adapters and PCR primers were removed using the cutadapt plugin. Quality score plots were inspected to determine appropriate trimming parameters for low-quality bases. The dada2 denoise-paired plugin included read filtering, denoising, merging into Amplicon Sequence Variants (ASVs), and chimera removal. ASVs were filtered to retain only those present in two or more samples via the filter-features plugin. Finally, taxonomic assignment was performed using a classifier trained on the customized 16S V3-V4 SILVA database (v. 138.2). Feature tables were rarefied prior to downstream diversity analyses.

The QIIME2 nested plugins were used to assess alpha diversity (Faith’s PD, Shannon and Evenness) and beta diversity (Jaccard and Bray–Curtis) metrics. Deeper exploration and statistical analysis were performed using R scripts implemented in the R-based tools MetaboAnalyst 6.0 (https://github.com/xia-lab/MetaboAnalystR, accessed on 22 April 2026) and MicrobiomeAnalyst 3.0 (https://github.com/xia-lab/MicrobiomeAnalystR, accessed on 22 April 2026).

### 2.9. Fecal Metabolome Untargeted Analysis

Volatile organic compounds (VOCs) were analyzed using a Clarus 680 gas chromatograph (Perkin Elmer, Beaconsfield, UK) fitted with an Rtx-WAX column (30 m × 0.25 mm internal diameter, 0.25 µm film thickness; Restek, Milan, Italy) and coupled to a Clarus SQ8MS mass spectrometer (Perkin Elmer, Beaconsfield, UK). Headspace solid-phase microextraction (HS-SPME) was carried out using a DVB/CAR/PDMS fiber (Supelco, Bellefonte, PA, USA), and subsequent GC–MS analysis [24]. Chromatograms were processed for peak identification using the 2008 National Institute of Standards and Technology (NIST) library. VOCs were identified based on a peak area exceeding 1,000,000 and a match probability of at least 85%, with manual verification of fragmentation patterns performed when necessary. 4-methyl-2-pentanol (final concentration 1 mg/L) served as the internal standard (IS). Quantification was achieved by interpolating relative peak areas against the IS signal, and results were reported as µg/g relative to the internal standard.

All analyses comparing pre- and post-intervention microbiota diversity metrics (alpha and beta diversity) and SCFA concentrations were prespecified in accordance with the study protocol’s primary and secondary outcomes. GPCS-stratified subgroup comparisons of microbiota composition, SCFA concentrations, and VOC profiles were likewise prespecified, consistent with the study’s a priori stratification design. In contrast, pairwise VOC comparisons across GPCS classes were exploratory and were not corrected for multiple testing; they are reported as hypothesis-generating findings and should be interpreted with caution. Similarly, the DAPC-based integrated discriminant analysis combining metataxonomic and biochemical variables was considered an exploratory post hoc analysis. No changes were made to the planned analyses after trial commencement.

### 2.10. Short Chain Fatty Acids Analysis and Quantification

Targeted short-chain fatty acid (SCFA) detection was conducted in IBS patients before and after trial intervention. Standard calibration curves were generated using pure reference standards of acetic, butyric, propionic, isobutyric, and isovaleric acids (Sigma-Aldrich, St. Louis, MO, USA) and an internal standard, i.e., 4-methyl-2-pentanol, at a final concentration of 1 mg/L was used. Total Ion Current (TIC) mode was employed to identify characteristic ions based on their specific mass-to-charge ratios for each short-chain fatty acid (SCFA). Subsequently, Selective Ion Monitoring (SIM) mode was utilized to quantify each compound. The relative peak areas of SCFAs in fecal samples were integrated, and their absolute concentrations (µg/g) were determined using the corresponding calibration curve equations.

## 3. Results

### 3.1. IBS Patient Cohort and Experimental Design

A total of 238 patients were assessed for eligibility between May 2022 and December 2023. Of these, 132 were excluded for the following reasons: 84 did not meet inclusion criteria (e.g., age outside the 18–65 range, IBS diagnosis not confirmed by Rome III/IV criteria, restricted diets, comorbidities), and 48 declined participations. A total of 106 patients with mild-to-moderate IBS were enrolled and commenced the 12-week structured exercise program (Supplementary Figure S1). Of the enrolled participants, 26 were excluded from the final analysis because they missed more than 20% of scheduled sessions, leaving 106 in the intention-to-treat analytical pool. Thus, our experimental design relies on 80 mild-to-moderate IBS patients who underwent a moderate-intensity aerobic exercise program and were assessed for clinical and microbiological profiling, using blood biochemical measurements in combination with metataxonomics and metabolomics profiles in a multi-omics approach.

For genus-level differential abundance (pre- vs. post-treatment and GPCS-stratified comparisons), the full set of 80 samples (both time points) was analyzed. For targeted SCFA and untargeted VOC profiling, a subset of 40 paired samples was included. All analyses were performed on the available data without imputation.

The included Supplementary Material file reports features relative to the enrolled patients evaluated in this study.

No significant adverse events were recorded during the 12-week intervention period, owing to continuous supervision by graduates in Science and Techniques of Preventive and Adapted Physical Activity. Heart rate was monitored in real time during each session; all participants maintained their target heart rate zone (60–75% of maximum heart rate). No participant was withdrawn from the study for safety reasons.

FastQ raw sequencing data are publicly available as NCBI BioProject under the following accession number: ID PRJNA1459045.

### 3.2. Alpha and Beta Diversity Metric Estimates

Based on per-sample assigned taxonomic features and after removing non-informative samples from rarefaction analysis, a total of 66 and 77 samples were retained to compute alpha and beta diversity metrics for the post- and pre-treatment groups, respectively (Supplementary Figure S2, panels A and B). When the alpha diversity was inspected, the Shannon entropy and the Faith’s Pd metrics did not show any statistically significant differences after pairwise Kruskal–Wallis correction.

Consistent with the lack of a sensible distinction between samples before and after the physical intervention, the unweighted UniFrac statistics for beta diversity did not resolve samples into distinct PCoA clouds, which, accounting for approximately 27% of the total distribution across three axes, largely overlapped (Supplementary Figure S3).

### 3.3. 16S Metataxonomics Variable Inspection

QIIME2 annotated taxa against the SILVA 138.2 database on a total of 80 samples before and after physical activity. Denoising was performed using the deblur nested plugin; the number of total retained reads ranged from roughly 15,000 to 200,000. A total of 11 phyla, 17 classes, 44 orders, 88 families, 223 genera, and 398 species were annotated and used in subsequent statistical analyses (Supplementary Material).

**Figure 1:**
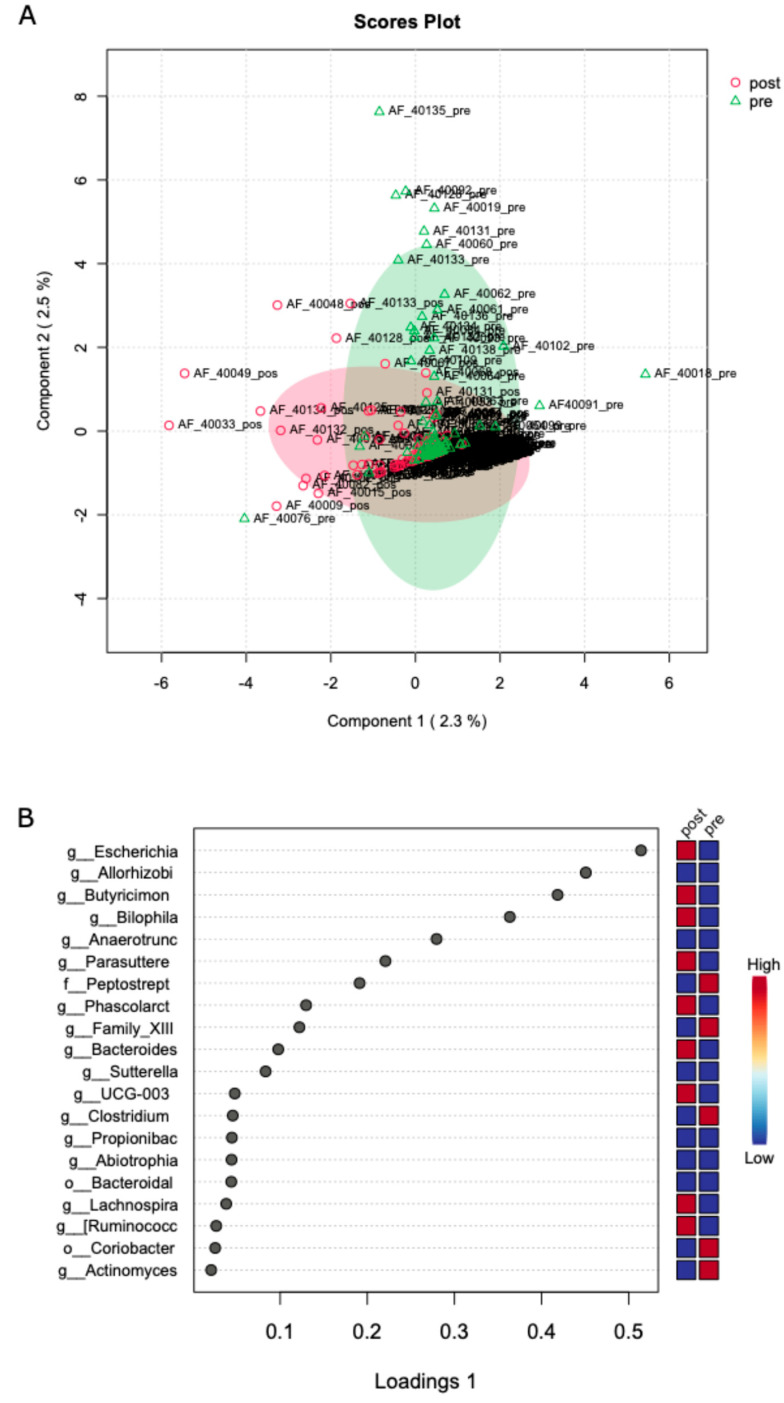
PLS-DA analysis between pre- and post-physical activity treated samples. Panel (**A**) PLS-DA plot. Panel (**B**) PLS-DA relative VIP scores.

In fact, based on the complete genus matrix, a first inspection was conducted using Partial Least Squares Discriminant Analysis (PLS-DA) combined with sparsity-based variable selection (sPLS-DA). The resulting clouds of pre- versus post-treatment samples were substantially overlapped (Figure 1A), with *Escherichia*, *Allorhizobium*, *Butyricimonas*, *Bilophila*, *Anaerotruncus*, *Parasutterella*, and *Peptostreptococcaceae* as the most contributing genera in the VIP score plot (Figure 1B).

**Figure 2:**
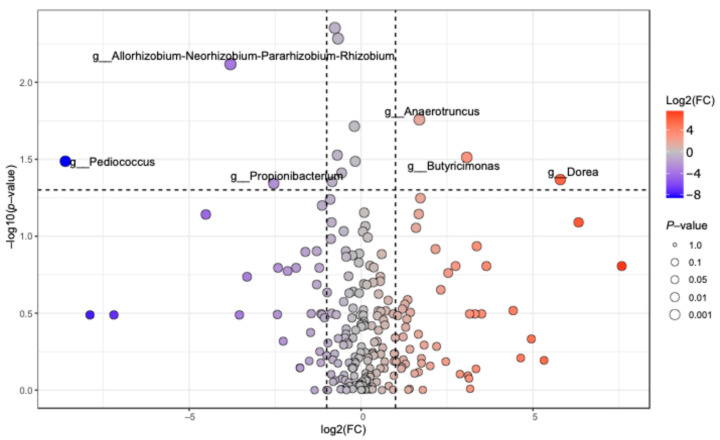
Volcano plot of before/after treatment group comparisons. The plot reports Log2 fold change on the X-axis versus −log10(*p*-value) on the Y-axis.

Pairwise comparison before and after the intervention was run to identify statistically significant changes in taxa relative abundances and revealed that *Allorhizobium–Neorhizobium–Pararhizobium–Rhizobium*, *Propionibacterium*, and *Pediococcus* decreased following the intervention, whereas *Dorea*, *Anaerotruncus*, and *Butyricimonas* increased (Figure 2 and Supplementary Table S1). 

### 3.4. GPCS Classification and DAPC-Based Posterior Grouping

A list of variables derived from blood measurements included biochemical parameters grouped into functionally defined classes, including glycemic/metabolic markers, renal function indices, liver function tests, lipid profile components, mineral and iron metabolism markers, inflammatory and protein biomarkers, thyroid and other endocrine hormones, vitamin status indicators, and hematological indices encompassing complete blood count and leukocyte differential profiles (Supplementary Material sheet “clinical”).

When blood biochemical values were used as input for Discriminant Analysis of Principal Components (DAPC), the pre- and post-treatment groups were not clustered separately. At the same time, when GPCSs before and after treatment were used as posterior-assigned groups, all samples collapsed in the center of the plot (Supplementary Figure S4), indicating insufficient discriminative power of biochemical variables alone.

**Figure 3:**
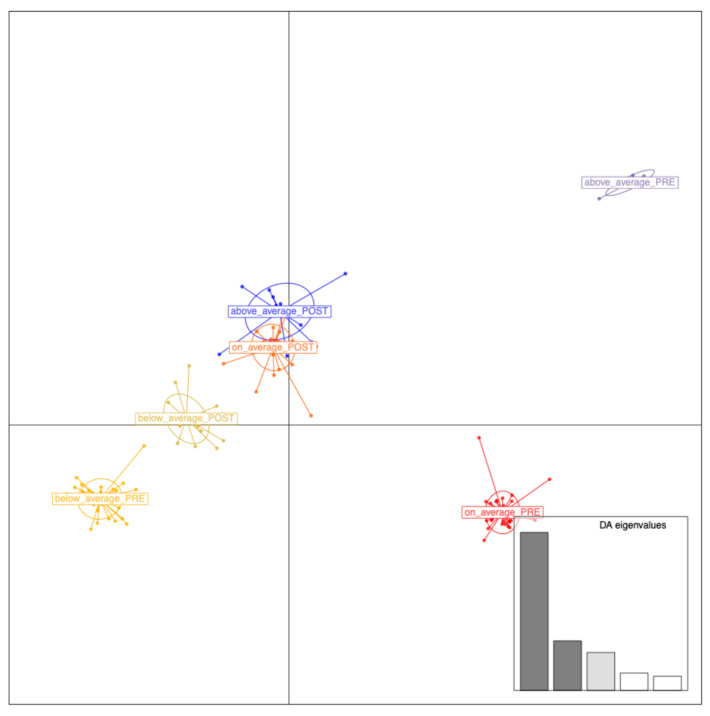
A posterior DAPC main plot based on the first two ranked eigenvalues.

To improve statistical resolution, metataxonomic data at the genus level were combined with biochemical clinical data, and the merged matrix was examined for clustering. As a result, subgroups showed clear separation, with above-average and below-average pre/post samples exhibiting the greatest linear distance along the first discriminant component (Figure 3).

The DAPC loading plot identified taxa-specific contributions to group clustering and specifically identified *Actinomyces*, *Parasutterella*, and *Roseburia* as the most impacting genera, alongside a specific clinical variable set including monocytes, neutrophils, and triglycerides (Supplementary Figure S5).

### 3.5. Inflammatory Marker Evaluation

Looking at the inspected inflammation markers, the only cytokine that resulted to be statically different in the two compared groups was TNF-alpha (Supplementary Figure S6).

### 3.6. Genus-Level Differential Abundance by GPCS Class

Statistically significant genera computed using the edgeR method were based on the GPCS grouping. The pairwise comparison of above- versus below-average GPCS samples after treatment revealed a decrease in *Collinsella* relative abundance in above-average samples, whereas an increase in the same genus was observed in below-average compared with on-average GPCS samples (Table 1; Supplementary Figure S7).

*Blautia* and *Subdoligranulum* relative abundances were increased in both above- and below-average GPCS samples compared with the on-average cluster.

### 3.7. SCFA Targeted GC/MS

**Figure 4:**
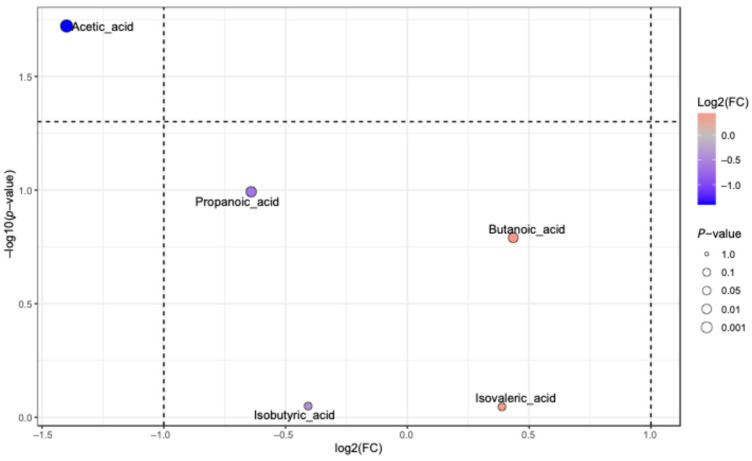
SCFA Volcano plot based on the GPCS above and below average sample groups. Two-group pairwise comparison of the inflammatory markers panel.

Pairwise group comparisons of physical activity treatment (T0 vs. T1) across 40 samples did not yield a statistically significant difference in SCFA concentrations. Although not corrected for multiple tests, the comparison of above- versus below-average GPCS groups was the only one showing a statistically significant increase in acetic acid concentrations after treatment (Figure 4). 

### 3.8. Untargeted VOC Profiling by GC/MS

**Figure 5:**
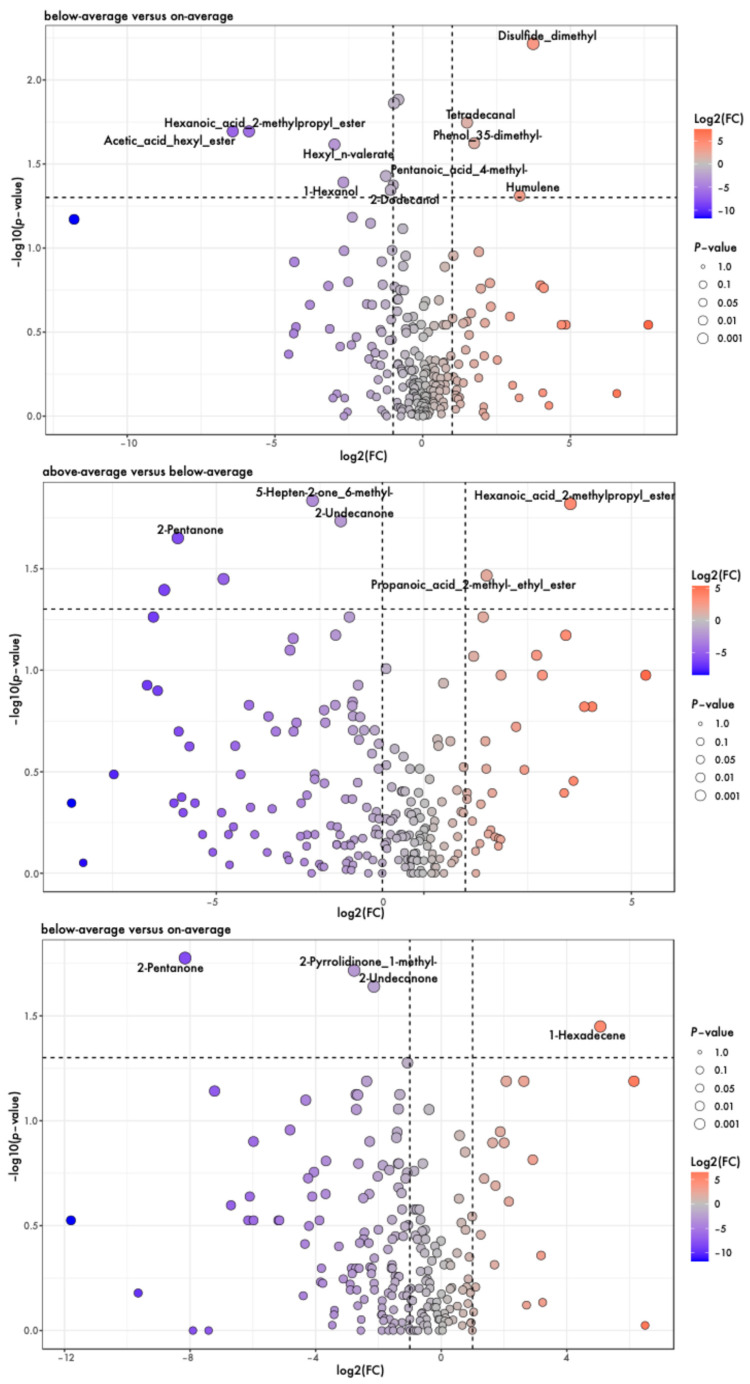
Volcano plot based on the GPCS class comparisons. Pairwise group comparison is indicated above each figure subpanel.

Pairwise comparisons between pre- and post-treatment samples (a total of 40) and stratified analyses by GPCS class were conducted on the complete matrix of volatile organic compound profiles (Figure 5).

The pairwise comparison between the above-average and on-average groups showed a significant decrease in the concentrations of 2-pentanone, 1-methyl-2-pyrrolidinone, and 2-undecanone, along with a significant increase in 1-hexadecene.

On the other hand, the comparison between the above-average and below-average groups showed reduced levels of 5-hepten-2-one (6-methyl-), 2-undecanone, 2-pentanone, 3,5-dimethylphenol, and α-farnesene in the above-average group, whereas hexanoic acid 2-methylpropyl ester and propanoic acid 2-methyl-ethyl ester exhibited increased concentrations.

Compared with the on-average GPCS group, the below-average group showed significantly lower concentrations of hexyl acetate, hexanoic acid 2-methylpropyl ester, hexyl n-valerate, 1-hexanol, 4-methylpentanoic acid, and 2-dodecanol, whereas tetradecanal, 3,5-dimethylphenol, humulene, and dimethyl disulfide were more abundant. 

## 4. Discussion

Using an integrated multi-omics approach, this study aimed to evaluate the effects of a structured moderate aerobic exercise program in a cohort of 80 patients with mild-to-moderate IBS, with *a priori* stratification based on the GPCS. Three principal findings emerge. First, moderate aerobic exercise does not induce a global restructuring of the gut microbiota but instead selectively modulates functionally relevant bacterial taxa involved in short-chain fatty acid (SCFA) production. Second, standard biochemical parameters fail to discriminate patient subgroups in response to the intervention, whereas the GPCS enables clear stratification. Third, GPCS-based stratification identifies distinct microbial and metabolomic response trajectories, suggesting that baseline physical capacity may, at least in part, contribute to determining exercise responsiveness in this population, potentially more than conventional blood biomarkers.

The absence of significant changes in alpha (Shannon, Faith’s PD) and beta (unweighted UniFrac) diversity is consistent with previous reports on moderate exercise interventions in non-athletic populations [25,26]. Microbial diversity is a relatively stable ecosystem property, more responsive to chronic or high-intensity perturbations than to moderate, time-limited interventions [27]. In this context, stability in diversity should not be construed as the absence of effect, but rather as evidence of subthreshold functional adaptation in the taxonomic assignments.

Despite this evidence, pre–post comparisons revealed significant shifts in specific taxa. Increases in *Dorea*, *Anaerotruncus*, and *Butyricimonas* are biologically consistent with enhanced fermentation of complex substrates and increased SCFA synthesis. *Dorea* is a saccharolytic genus associated with carbohydrate fermentation and acetate production [28,29], whereas *Anaerotruncus* and *Butyricimonas* are recognized as butyrate producers. Butyrate is central to epithelial barrier integrity, mucosal immune regulation, and attenuation of low-grade inflammation—key processes in IBS pathophysiology [30,31,32,33]. Conversely, reductions in environmental taxa, such as the *Allorhizobium–Neorhizobium–Pararhizobium–Rhizobium* group and *Pediococcus*, are consistent with exercise-induced physiological changes, including accelerated intestinal transit and altered substrate availability [34,35]. Causal directionality cannot be established in the absence of a concurrent control group.

A pivotal contribution of this work is demonstrating that GPCS, despite not stratifying groups by standard biochemical parameters, identifies subpopulations with significantly distinct microbial and metabolomic responses. Patients stratified by baseline GPCS exhibited divergent adaptation trajectories, indicating that response heterogeneity is biologically structured rather than stochastic.

Integrated discriminant analysis (DAPC), combining taxonomic and clinical variables, revealed clear separation between GPCS groups, with the above-average subgroup showing the greatest displacement along the primary discriminant axis. Key contributors to this separation included *Actinomyces*, *Parasutterella* and *Roseburia*, alongside clinical parameters such as monocytes, neutrophils and triglycerides. *Roseburia*, a well-characterized butyrate producer, has been associated with reduced intestinal permeability and systemic inflammation [32,36,37], while *Parasutterella* is implicated in bile acid and lipid metabolism and is sensitive to lifestyle modulation [38]. Their contribution to group separation suggests that baseline physical capacity may define the gut ecological niches amenable to exercise-induced modulation.

Comparative analyses across GPCS classes further identified differential variation in *Collinsella*, *Blautia*, and *Subdoligranulum*. *Blautia*, a producer of acetate and butyrate with recognized anti-inflammatory properties, is inversely associated with systemic inflammation and metabolic dysbiosis [39]. Although exploratory, its increased abundance in both extreme GPCS groups (above- and below-average) relative to the intermediate group suggests potentially distinct, non-linear microbial response trajectories linked to baseline physical phenotype. *Subdoligranulum* shares similar metabolic features and is likewise associated with butyrate production and mucosal homeostasis [40].

It should also be clarified that the direction of the relationship between GPCS and gut microbiota responsiveness cannot be established from the present data. Two interpretations are equally plausible. Lower physical capacity in IBS patients may reflect a primary fitness deficit that constrains physiological plasticity and limits the gut ecosystem’s capacity to mount a coherent adaptive response to exercise. Alternatively, reduced physical capacity may be a downstream consequence of IBS symptom burden: patients who experience chronic pain, urgency, or fatigue tend to restrict their activity, and the same symptom profile that drives deconditioning may independently predict a blunted response to any intervention. Under this second reading, GPCS would function as a proxy for symptom severity rather than an independent biological modifier of exercise responsiveness.

Both mechanisms are biologically plausible and are not mutually exclusive. Disentangling them requires a prospective design that includes serial GPCS assessments at multiple time points, paired with systematic symptom tracking, so that changes in physical capacity and symptom burden can be examined as parallel and potentially interacting trajectories rather than as static baseline characteristics.

At the metabolomic level, total SCFA concentrations did not change significantly across the cohort. However, the above-average GPCS subgroup showed a post-intervention increase in acetate levels. Although not corrected for multiple testing and therefore to be interpreted cautiously, this finding aligns with the observed enrichment of saccharolytic taxa and suggests that individuals with higher baseline physical capacity may derive greater metabolic benefit from identical exercise protocols [41].

Untargeted volatile organic compound (VOC) profiling revealed differential remodeling across GPCS stratification. In above-average individuals, reductions in compounds such as 2-pentanone and 2-undecanone, ketones and aldehydes associated with dysbiotic anaerobic fermentation and oxidative stress, combined with increased medium-chain esters, indicate a shift toward a more efficient and less pro-inflammatory microbial metabolism. VOCs represent a particularly promising metabolomic window in IBS, as direct products of microbial fermentation with potential for non-invasive detection.

An apparently counterintuitive finding was the increase in TNF-α observed in the above-average group following intervention (not corrected for multiple testing and thus exploratory), in the absence of changes in other systemic inflammatory markers (CRP, IL-6, IL-8, IL-10). This pattern warrants contextual interpretation. TNF-α plays a dual role, functioning not only as a pro-inflammatory mediator but also in tissue repair, immune priming and epithelial turnover [42]. In the absence of broader inflammatory activation and alongside a microbial and metabolomic profile indicative of reduced oxidative stress, this finding is more consistent with adaptive immune activation, previously described as exercise-induced immune priming, than with inflammation exacerbation [43].

From a clinical perspective, the key observation is that standard biochemical and inflammatory markers, along with intestinal permeability measures, do not distinguish response subgroups, whereas GPCS does. This suggests that baseline physical capacity, often overlooked in gastroenterological practice, may provide prognostic information not captured by routine laboratory assessments.

Operationally, these findings indicate that, in patients with mild-to-moderate IBS, a standardized moderate aerobic exercise program may be an effective stand-alone therapeutic strategy, particularly for those with above-average baseline GPCS. These patients appear to exhibit greater physiological plasticity, leading to more coherent microbial and metabolomic adaptations. In contrast, individuals with below-average GPCS show distinct, albeit not absent, response profiles, suggesting that tailored, progressive exercise prescriptions may optimize outcomes in this subgroup.

This framework aligns with emerging paradigms in personalized exercise medicine and functional approaches to gastrointestinal disorders [44,45]. The use of GPCS as a pre-intervention stratification tool may be a practical step toward standardizing exercise prescription for IBS, reducing the variability that has historically limited its clinical adoption.

Above all, the observed biological changes, including enrichment of SCFA-producing taxa, reduction in dysbiosis-associated VOCs, and increased acetate in the above-average subgroup, align with symptom improvements reported in prior studies from our group using the same exercise protocol. Although the present study did not include prospective symptom assessments, this convergence supports the biological plausibility of the findings and warrants validation in future studies that integrate clinical endpoints.

Importantly, symptomatic data from the same cohort reported in companion studies from our group [15,16] showed that GPCS-based stratification was associated with differential improvements in IBS symptom severity (IBS-SSS) and psychological distress, with above-average GPCS patients showing greater overall responsiveness. The microbiota and metabolomic findings reported here (enrichment of SCFA-producing taxa and reduction in dysbiosis-associated VOCs in the above-average subgroup) may represent part of the biological mechanism underlying those differential symptom trajectories. Formal integration of symptom endpoints with omics data in a prospectively designed, adequately powered study remains an essential validation step before GPCS can be adopted as a clinical stratification tool.

Several limitations should be acknowledged. The absence of a sedentary control group precludes causal inference, and leaves open the possibility of temporal confounding. The cohort was limited to patients with mild-to-moderate IBS recruited from a tertiary-care center in southern Italy, introducing potential referral bias and limiting generalizability to primary care settings, severe IBS phenotypes, and individual IBS subtypes [42,43]. The single-center design and the demographic homogeneity of the population, largely Caucasian adults following a Mediterranean dietary pattern, further restrict the applicability of microbiota and metabolomic findings to populations with different ethnic, dietary, and geographic backgrounds. Some metabolomic analyses were not adjusted for multiple comparisons and should therefore be considered exploratory.

A ceiling effect in the above-average GPCS patients cannot be entirely ruled out. This subgroup shows greater post-intervention changes in SCFA levels and more coherent VOC remodeling. As plausible biological explanation, the above-average GPCS phenotype likely reflects habitually higher activity over superior physiological plasticity, including more efficient gut fermentation capacity, and a greater metabolic flexibility. In this framework, the gut microbiome of a more physically capable individual may be better positioned to mount a structured adaptive response to a defined aerobic stimulus, not because the stimulus is novel, but because the host ecosystem is more amenable to coherent modulation.

We also acknowledge how dietary intake was not prospectively tracked during the intervention period, and we recognize this to be a genuine limitation. Exercise-induced changes in appetite or spontaneous food choices—particularly increasing fiber consumption—could contribute to the observed SCFA shifts and cannot be ruled out as partial confounders.

One pattern worths to be reported is the increase in acetate observed in the above-average GPCS subgroup only. Although a more uniform response across both subgroups can be expected, the group-differential pattern observed is more consistent with phenotype-specific metabolic plasticity.

Finally, the relatively short duration of the intervention does not allow assessment of long-term sustainability or dose–response relationships. Future multicenter studies including more diverse populations, IBS subtypes, and exercise modalities are needed to strengthen external validity and confirm the clinical utility of GPCS-based stratification.

Data confirmation will require future studies incorporating prospective dietary assessment, based on validated food frequency questionnaires or controlled dietary conditions, in parallel with the exercise intervention.

Nonetheless, the longitudinal within-subject design is a methodological strength for capturing individual physiological plasticity, minimizing inter-individual variability, and reconstructing patient-specific adaptation trajectories, information not accessible through cross-sectional or between-group designs.

At present date, GPCS should be treated as a hypothesis-generating stratification tool, not a clinical instrument ready for routine gastroenterological practice. Before it can be seriously considered for clinical adoption, three conditions need to be met. First, its performance must be validated in independent cohorts. Second, scoring norms require standardization across populations varying in sex, age, and ethnic background. Third, and most importantly, its capacity to predict outcomes that matter to patients, rather than biological trajectories alone, must be directly demonstrated.

The present study establishes proof of concept for the GPCS’s biological discriminative capacity in the context of exercise-induced gut microbiota remodeling in IBS. The step from biological proof of concept to clinical utility is real and non-trivial, and we regard it as the primary objective for the prospective research this work is intended to motivate.

Future randomized controlled trials that incorporate prospective GPCS stratification, active control groups, and longitudinal multi-omics profiling are needed to formally validate GPCS as a tool for personalized exercise prescription in IBS.

## 5. Conclusions

This multi-omics study demonstrates that a moderate aerobic exercise program selectively modulates the gut microbiota and metabolic profile in patients with mild to moderate IBS, characterized by enrichment of short-chain fatty acid (SCFA)-producing taxa and remodeling of the volatile organic compound (VOC) landscape toward a more efficient microbial metabolic state. Crucially, this modulation is not homogeneous across cohorts but is structured by baseline physical capacity as assessed by the GPCS. Given that conventional biochemical parameters fail to discriminate responder subgroups, the GPCS emerges as a functional stratification tool with direct clinical applicability, enabling identification of IBS patients likely to benefit from exercise as a standalone therapeutic intervention, without the need for adjunctive pharmacological or dietary measures. Collectively, these exploratory findings provide a rationale for prospective controlled studies to validate exercise prescription as a structured component of personalized IBS management.

## Figures and Tables

**Table 1:** Statistically significant genera by GPCS class comparison (edgeR method) GPCS class comparison.

GPCS Class Comparison	log2FC	logCPM	*p*-Value	FDR
Below average vs. on-average				
* B* *lautia*	−4.46	20.50	8.16 × 10^−16^	6.53 × 10^−15^
* Subdoligranulum*	−5.61	20.33	2.09 × 10^−15^	8.38 × 10^−15^
* Collinsella*	+1.78	17.25	4.46 × 10^−10^	1.19 × 10^−9^
Above vs. below average				
* Collinsella*	−1.48	17.25	3.98 × 10^−5^	3.18 × 10^−4^
Above average vs. on-average				
* Blautia*	−4.44	20.50	7.47 × 10^−13^	5.97 × 10^−12^
* Subdoligranulum*	−5.41	20.33	5.00 × 10^−12^	2.00 × 10^−11^

## Data Availability

All data are available within the paper or as Supplementary Materials. FastQ raw sequencing data are publicly available as NCBI BioProject under the following accession number: ID PRJNA1459045.

## Supplementary Materials

The following supporting information can be downloaded at: https://www.mdpi.com/article/10.3390/nu18121972/s1, Supplementary Figure S1. CONSORT flowchart diagram. Enrolled, excluded and final cohort participants relative to the study. Supplementary Figure S2. Panel (A) Shannon entropy box plots relative to pre- and post-treated samples. Kruskal–Wallis multiple test corrected test on Shannon alpha diversity computed metric. Panel (B) Shannon H index with pairwise Kruskal–Wallis relative *p*-value and corrected q-value. Supplementary Figure S3. PCoA of beta diversity using the unweighted UniFrac metric. Unweighted unifrac distance values for beta diversity have been used to compute a three-axis PCoA. Supplementary Figure S4. A posterior DAPC plot based on blood biochemical values. DAPC plot superimposing group belonging based on biochemical clinical values. Supplementary Figure S5. DAPC loading plot based on clinical and microbiological variables. An arbitrary threshold of 0.01 was chosen to highlight the most impactful statistical variables. Supplementary Figure S6. Violin plots reporting the distribution of samples based on TNF-alpha inflammatory marker values. Raw and normalized concentrations in the above-average and below-average groups after the physical activity treatment were reported as violin plots. Supplementary Figure S7. Statistically significant genera from GPCS pairwise group comparisons. Differential abundance analysis of 16S genera using edgeR, applying a negative binomial model to normalized count data. Each point represents a microbial taxon (feature). Point size is proportional to the logarithm of the total abundance of the taxon across all samples. The y-axis shows −log10(*p*-value), where larger values indicate greater statistical significance of differential abundance between groups. Taxa exhibiting significant differences between conditions are displayed, with statistical significance expressed as −10 log_10_(*p*-value). The upward-facing triangle shape indicates a positive fold change, whereas the downward-facing triangle denotes a negative fold change. Panel (A) Below- vs. on-average, Panel (B) Above- vs. below-average, Panel (C) Above- vs. on-average Supplementary Table S1. Statistically significant genera identified based on pairwise comparison of pre- and post-treatment groups (edgeR method). Supplementary Material. The excel file includes sequencing analysis statistics and denoising on sample reads (sheets “demux_filtered_stats”, “paired_end_demux”, “deblur_stats”); taxa annotation at different taxonomics l level (sheets “phylum”, “class”, “order”, “family”, “genus”, “species”); clinical biochemical blood parameters (“clinical”); VOC untargeted and targeted analysis (sheets “untargeted_VOCs”, “targeted_VOCs”); antropometric, GPCS, bioimpedance variables (sheets “antropometry”, “GPCS”, “bioipedance analysis”); IBS-SSS, intestinal barrier integrity and interleukins parameters (sheets “IBS-SSS”, “barrier integrity“, “IL_panel”).
