# Supplementary material for: GPCS Stratification of Exercise-Induced Gut Microbiota and Metabolome Remodeling in IBS: An Exploratory Multi-Omics Study
Source: Nutrients. 2026 Jun 18;18(12):1972. doi: 10.3390/nu18121972 (PMC13304523; doi:10.3390/nu18121972)
Supplement: Supplementary file 1 [file nutrients-18-01972-s001.zip › Supplementary Table S1.pdf]

Supplementary Table S1. Statistically significant genera identified based on pairwise comparison of pre- and post-treatment groups (edgeR method).

Genus	FC	log2(FC)	Corr. p-value	–log10(p)
Allorhizobium–Neorhizobium–Pararhizobium–Rhizobium	0.072	–3.80	0.0076	2.12
Anaerotruncus	3.24	1.69	0.0175	1.76
Butyricimonas	8.42	3.07	0.0307	1.51
Pediococcus	0.003	–8.60	0.0326	1.49
Dorea	55.49	5.79	0.0429	1.37
Propionibacterium	0.172	–2.54	0.0455	1.34
