# Supplementary figures and images for: GPCS Stratification of Exercise-Induced Gut Microbiota and Metabolome Remodeling in IBS: An Exploratory Multi-Omics Study
Source: Nutrients. 2026 Jun 18;18(12):1972. doi: 10.3390/nu18121972 (PMC13304523; doi:10.3390/nu18121972)

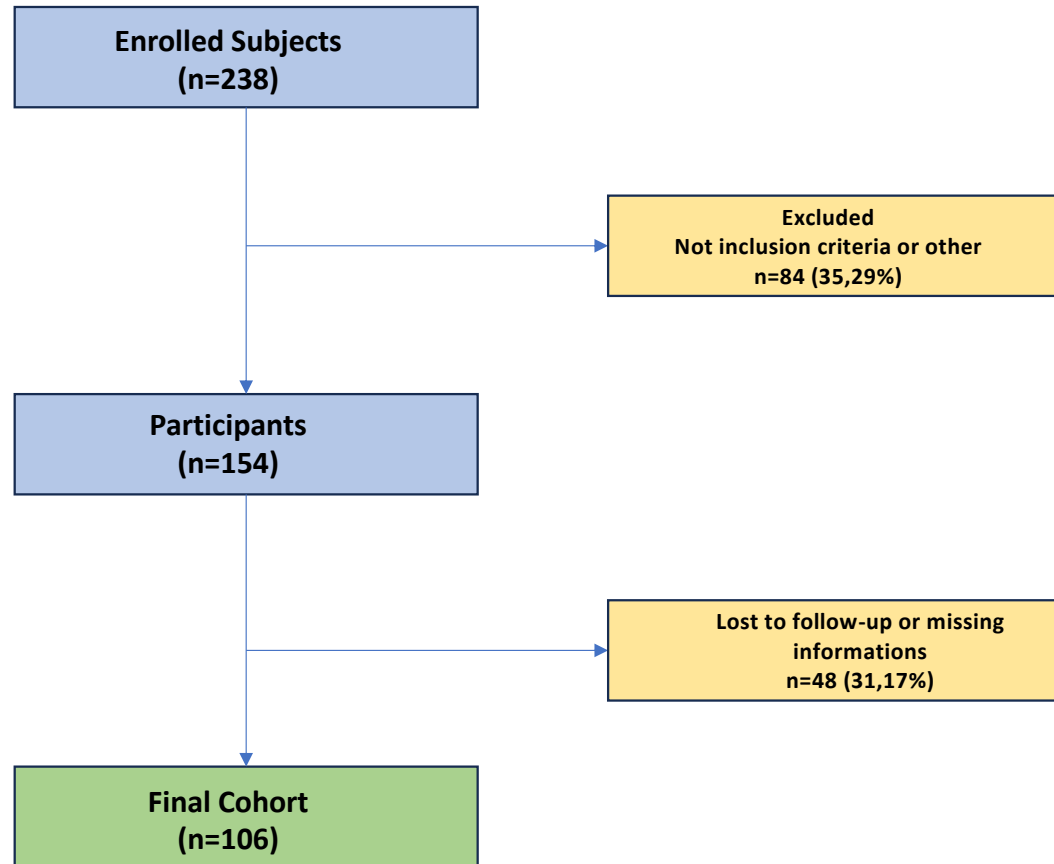

Supplement: Supplementary file 1 [file nutrients-18-01972-s001.zip › Supplementary_Figure_S1.pdf]

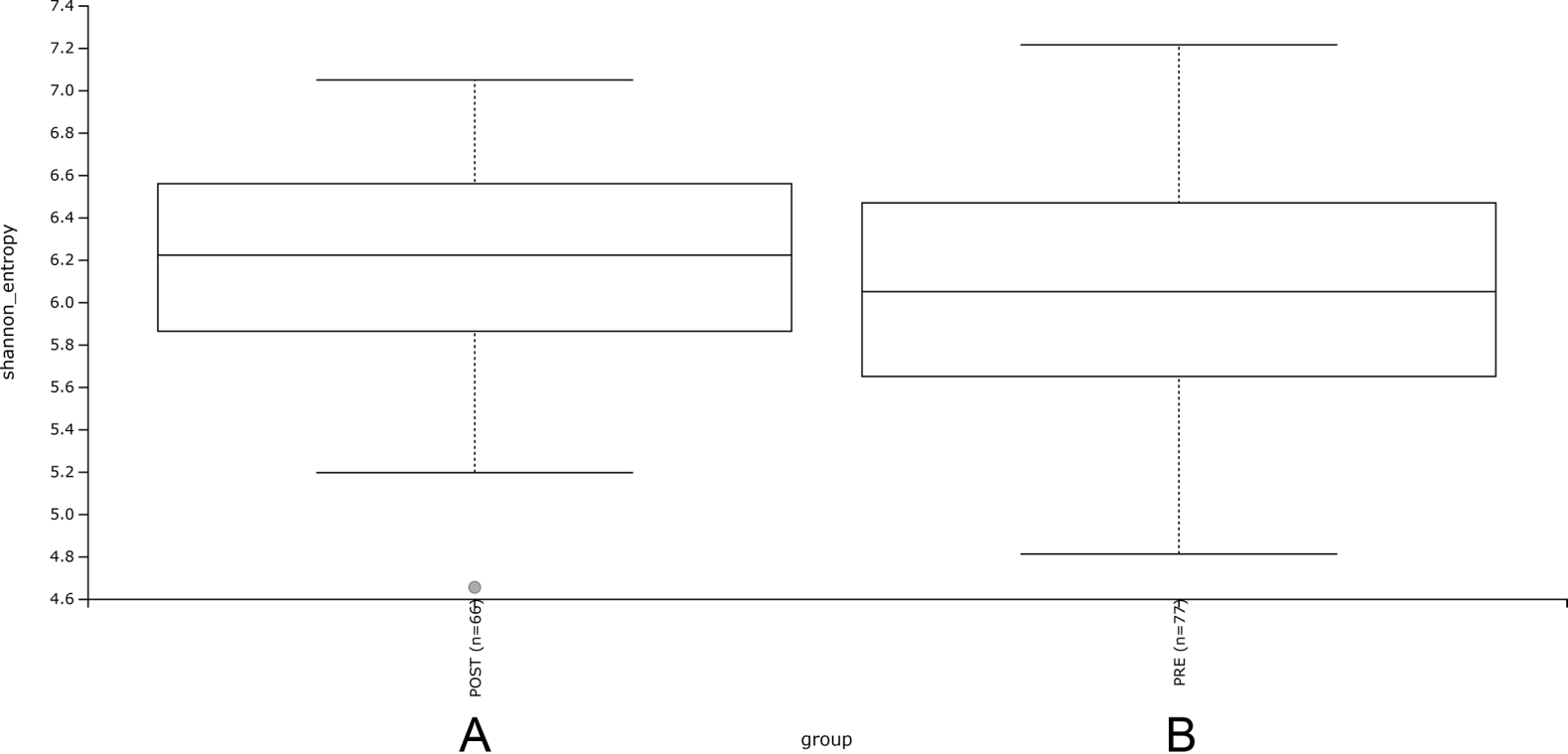

# Kruskal-Wallis (pairwise)

[Download CSV](#)

|             |            | H        | p-value  | q-value  |
|-------------|------------|----------|----------|----------|
| Group 1     | Group 2    |          |          |          |
| POST (n=66) | PRE (n=77) | 2.380313 | 0.122873 | 0.122873 |

Supplement: Supplementary file 1 [file nutrients-18-01972-s001.zip › Supplementary_Figure_S2.pdf]

Axis 2 (6.742 %)

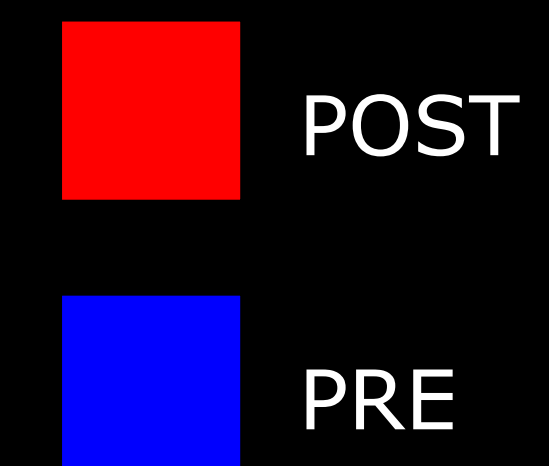

Axis 1 (15.42 %)

Axis 3 (4.629 %)

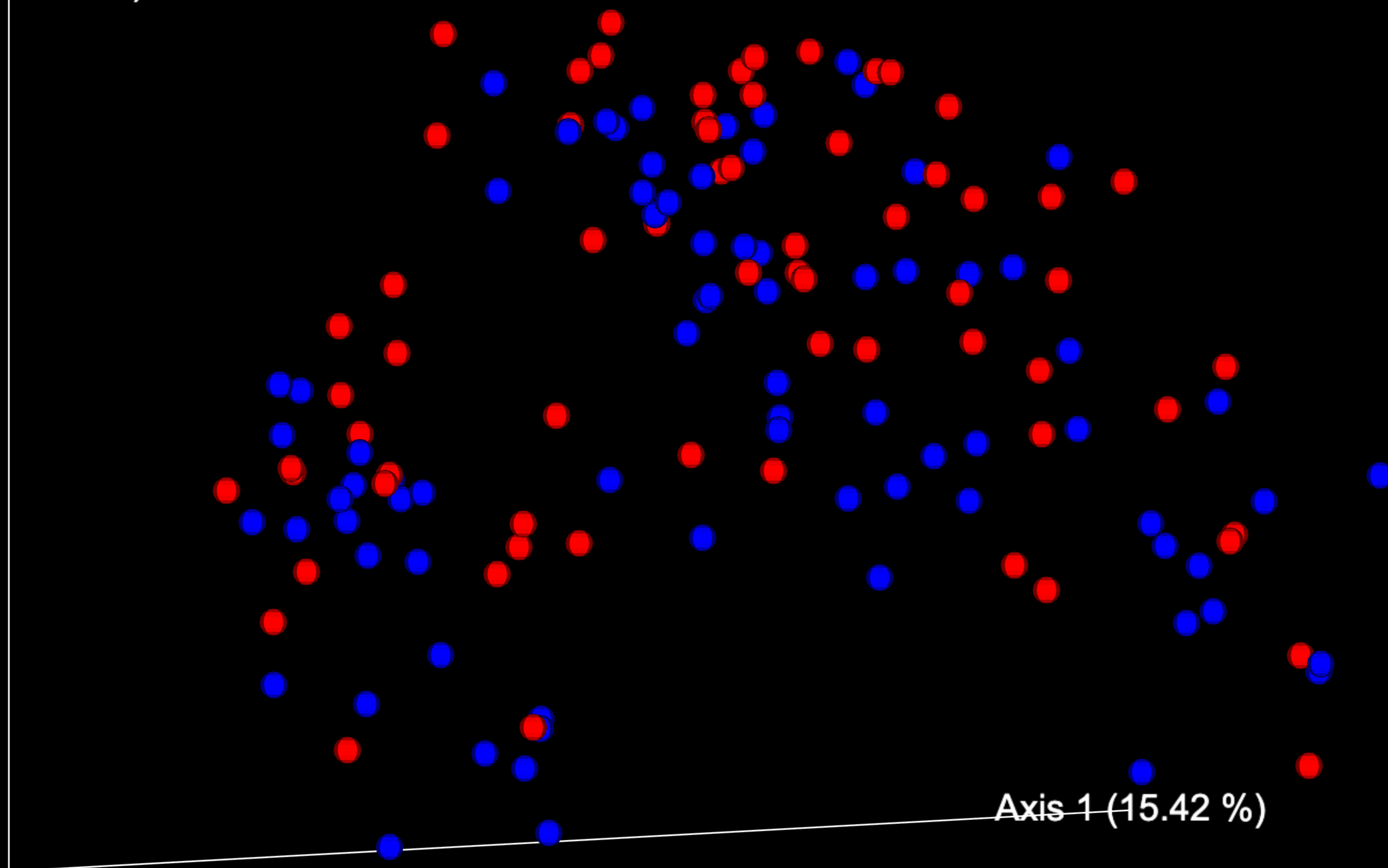

Supplement: Supplementary file 1 [file nutrients-18-01972-s001.zip › Supplementary_Figure_S3.pdf]

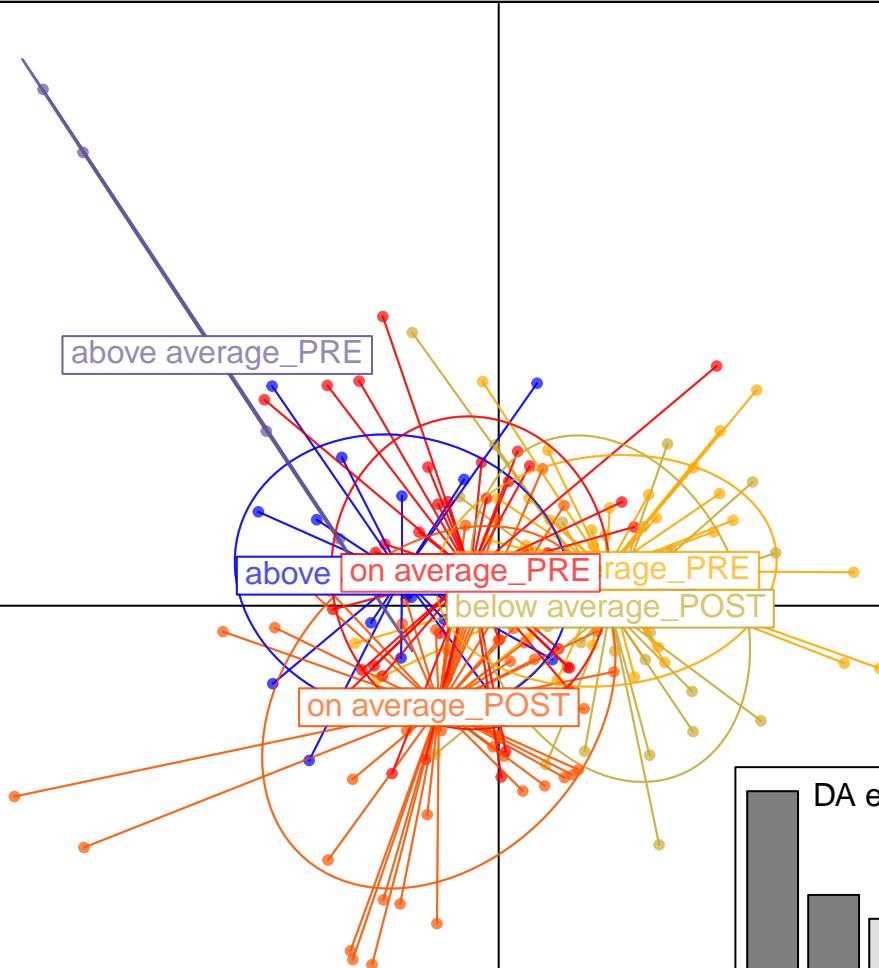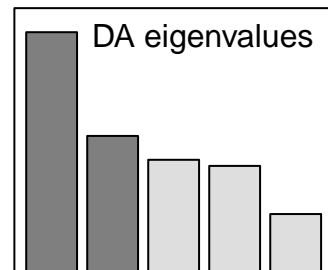

Supplement: Supplementary file 1 [file nutrients-18-01972-s001.zip › Supplementary_Figure_S4.pdf]

# Loading plot

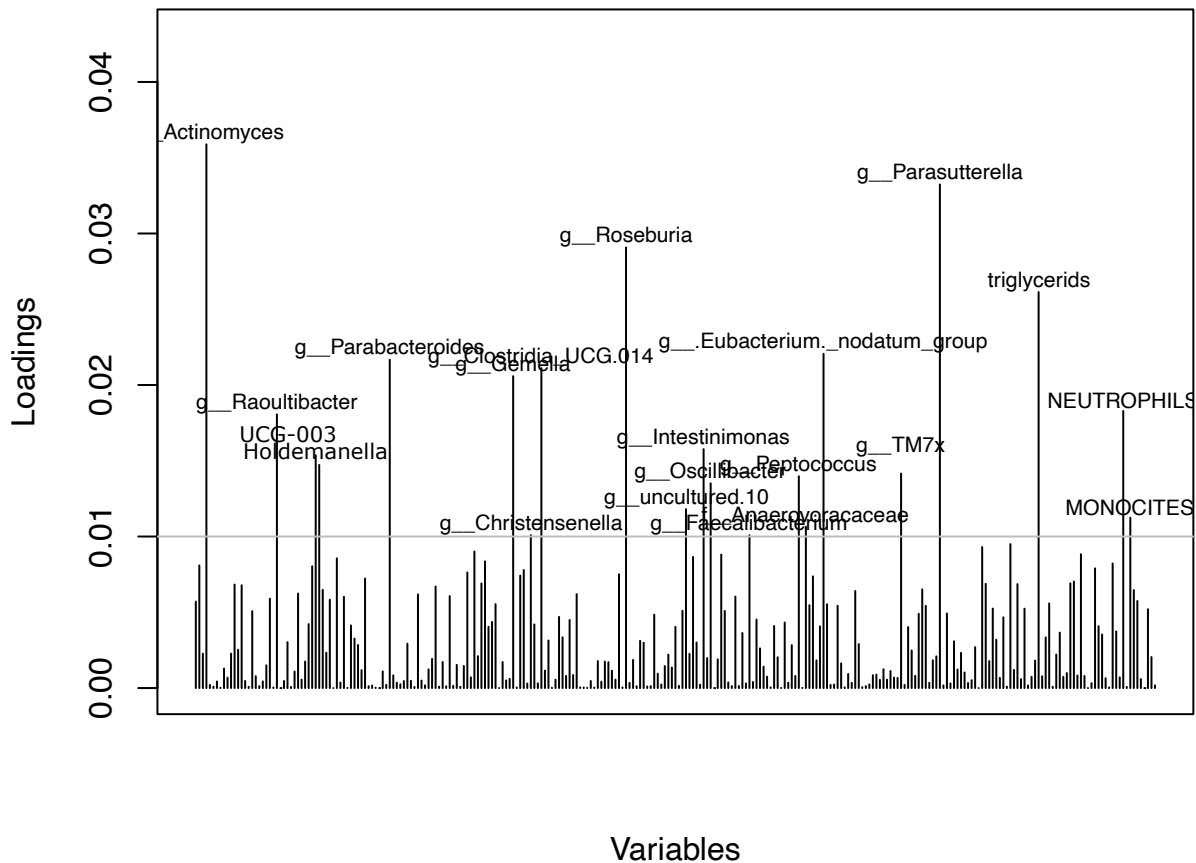

Supplement: Supplementary file 1 [file nutrients-18-01972-s001.zip › Supplementary_Figure_S5.pdf]

# TNF-alpha

## Original Conc.

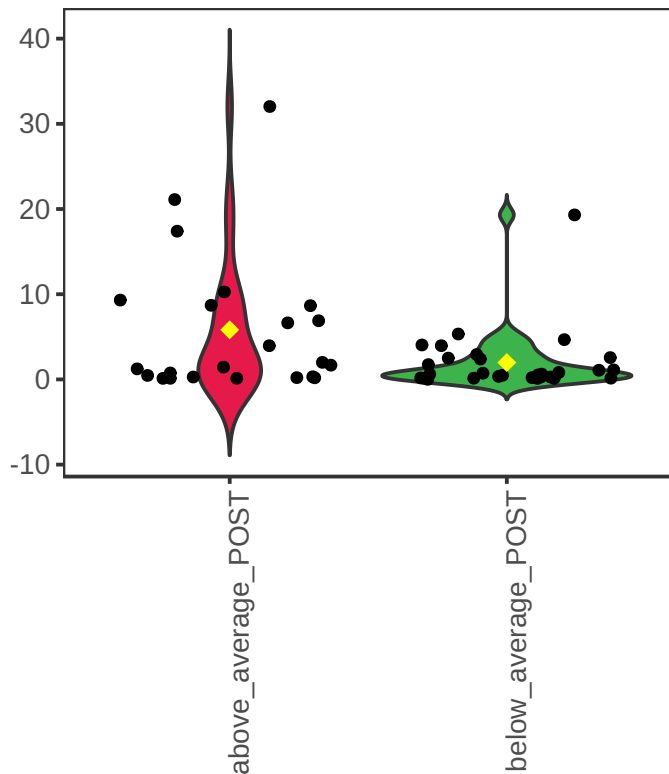

## Normalized Conc.

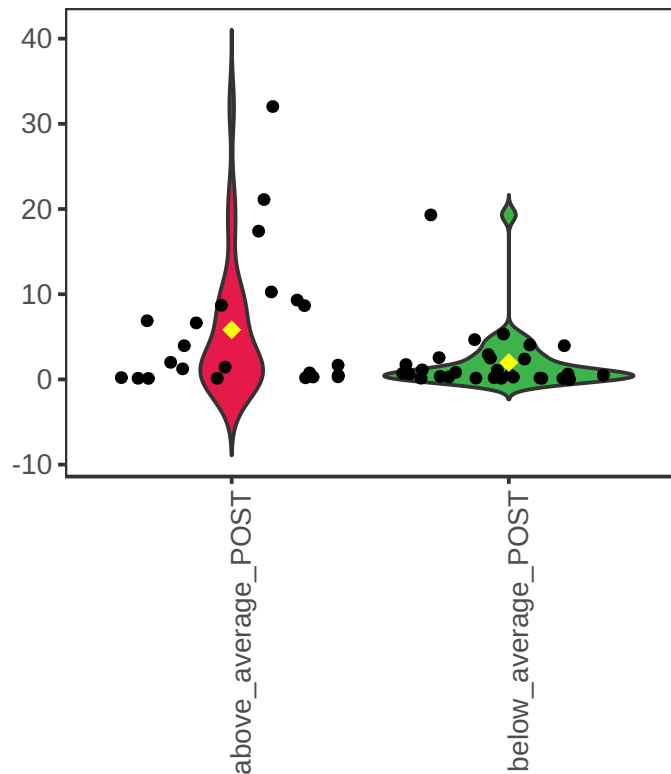

Class

Supplement: Supplementary file 1 [file nutrients-18-01972-s001.zip › Supplementary_FIgure_S6.pdf]

# A Below-average vs on-average

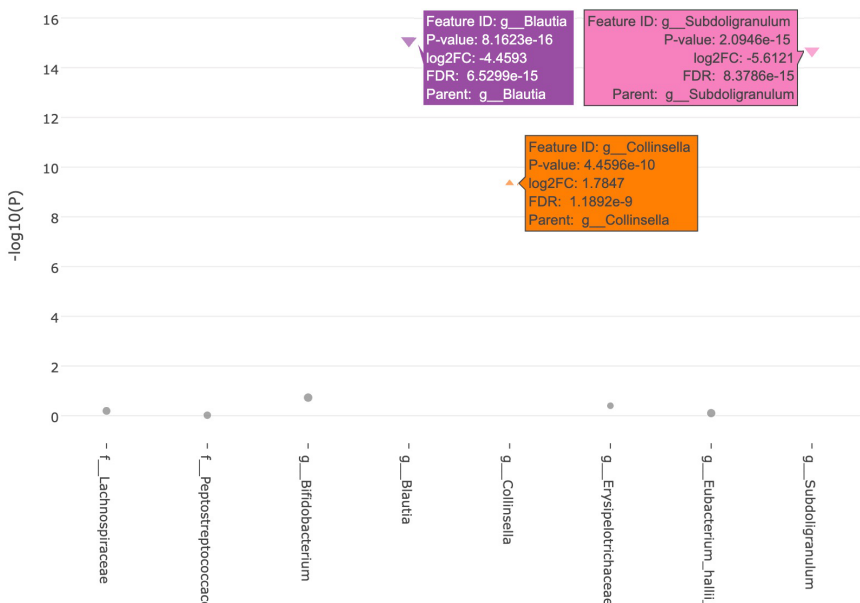

# B Above-average vs below-average

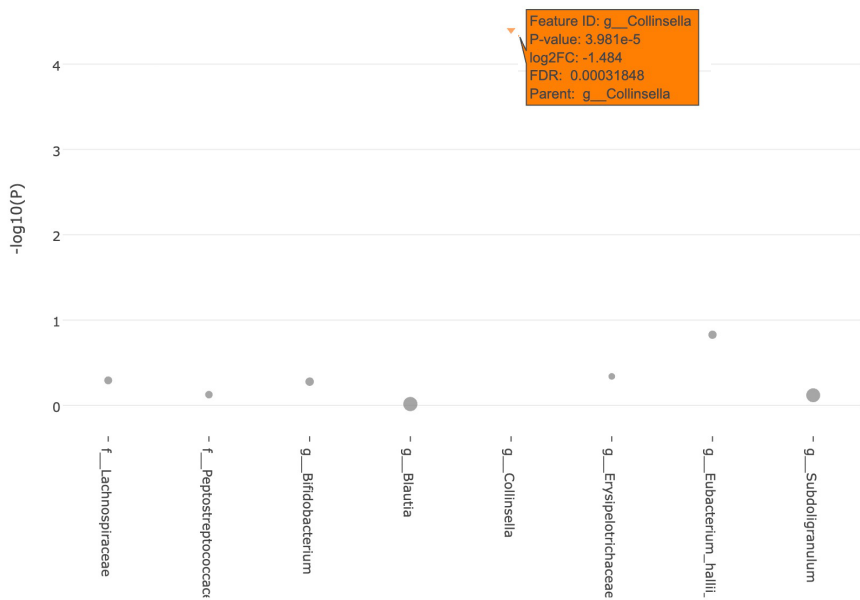

# C Above-average vs on-average

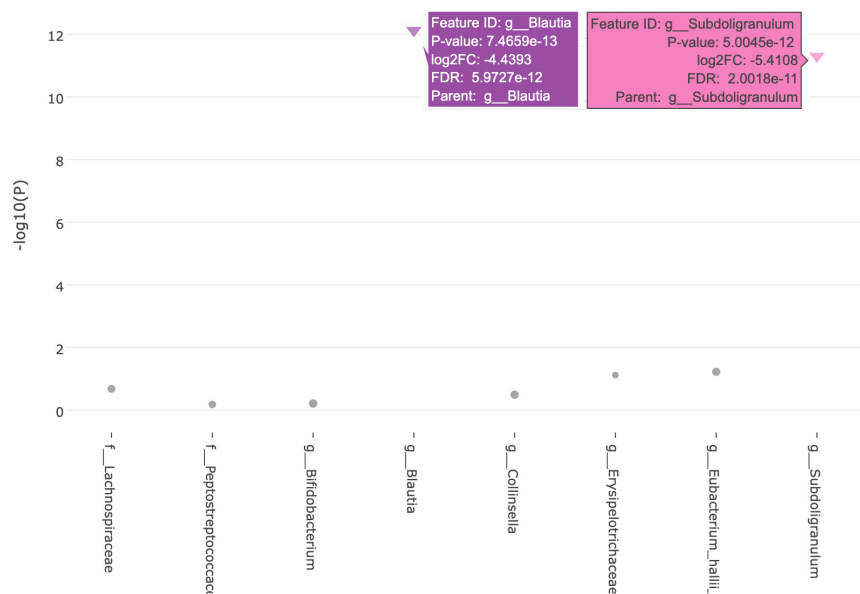

Supplement: Supplementary file 1 [file nutrients-18-01972-s001.zip › Supplementary_Figure_S7.pdf]
